# PreserFlo™ MicroShunt Versus Ab Externo Canaloplasty in Patients With Moderate to Advanced Open-Angle Glaucoma: 12-Month Follow-Up of a Single-Center Retrospective Study

**DOI:** 10.7759/cureus.35185

**Published:** 2023-02-19

**Authors:** Kirsten Julia Habbe, Markus Kohlhaas, Sofia Fili

**Affiliations:** 1 Department of Ophthalmology, St. Johannes Hospital, Dortmund, DEU

**Keywords:** preserflo™ microshunt, canaloplasty, intraocular pressure, moderate to advanced glaucoma, less invasive glaucoma surgery

## Abstract

Purpose

To evaluate and compare the efficacy and safety of the PreserFlo MicoShunt and the canaloplasty in patients with moderate to advanced glaucoma.

Methods

In this retrospective study, 300 patients with moderate to advanced glaucoma underwent either the implantation of the PreserFlo™ MicroShunt (group A) or a canaloplasty (group B). All patients underwent regular follow-up examinations in our department every two days, every two weeks, and every three, six, and 12 months postoperatively. Examinations included measurement of the best corrected visual acuity (BCVA), slit-lamp biomicroscopy of the anterior and posterior segments, intraocular pressure (IOP) measurement using Goldmann applanation tonometry, visual field perimetry, and measurement of the endothelial cell density (ECD). Efficacy was shown by the absolute and qualified success rates calculated with the Kaplan-Meyer analysis.

Results

In group A, IOP was significantly reduced at 12 months (13.37 ± 3.94 mmHg, p≤ 0.01) postoperatively in comparison to baseline (23.47 ± 8.39 mmHg). In group B, there was also a reduction in IOP at 12 months (14.32 ± 3.59 mmHg, p≤ 0.01) in comparison to the baseline (18.86 ± 5.82 mmHg). Comparing both groups, the IOP of patients who received the PreserFlo™ MicroShunt was significantly lower than the IOP of patients receiving canaloplasty after 12 months (p=0.049).

Patients in both groups were treated with significantly fewer topical agents after 12 months (group A: baseline = 2.53±1.56; 12 months: 0.43 ± 0.83, p≤0.01; group B: baseline 2.62 ± 0.87; 12 months: 1.52 ± 0.99, p≤0.01). Patients receiving the PreserFlo™ MicroShunt applied significantly fewer topical agents than patients who underwent canaloplasty (p≤ 0.01).

One year after surgery, the cumulative probability of absolute success was 81.33% in group A and 14.67% in group B. After one year, the cumulative probability of qualified success was 93.33% in group A and 82.00% in group B.

Conclusion

The PreserFlo™ as well as the canaloplasty offer many advantages and only a few disadvantages for patients with glaucoma. However, the respective patient’s history and individual risk profile play an important role in the decision of the glaucoma specialist regarding the most appropriate surgical treatment for each patient. Patients with a high risk of conjunctival scarring and postoperative complications may benefit more from a canaloplasty, whereas patients who need a lower average IOP and show intolerance to any topical agents may require the PreserFlo™ implantation.

## Introduction

Glaucoma is the main cause of irreversible blindness, with a continually rising prevalence, not only in Europe but also worldwide [1]. The management of glaucoma focuses on reducing intraocular pressure (IOP) [2]. Conservative medical therapy is currently the first-line treatment for patients with glaucoma [3]. In cases of insufficient reduction of IOP under conservative therapy, surgical intervention is required to avoid further progression of optic nerve damage [2]. Trabeculectomy is widely considered the gold standard treatment but is associated with the risk of severe postoperative complications such as overfiltration with bulbous hypotony, blebitis, conjunctival dehiscence, and leakage [4, 5].

In the last decade, surgical methods have been developed that aim to reduce the IOP sufficiently with less invasiveness and a lower risk of complications, like the so-called Less Invasive Glaucoma Surgery (LIGS) [6-8]. Many micro-implants have been produced that use subconjunctival space as an alternative drainage path to lower intraocular pressure [5, 8, 9].

The PreserFlo™ MircoShunt (Santen Inc., Miami, FL) is an 8.5 mm long LIGS device with an outer diameter of 350 µm and 70 µm lumen [10]. It is composed of poly (styrene-block-isobutylene-block-styrene) ("SIBS") [10]. The PreserFlo MircoShunt is implanted into the anterior chamber and forms a posterior filtration bleb under the conjunctiva and Tenon’s capsule [4, 10].

A different approach to surgically reducing the IOP is, for example, canaloplasty, which is a non-penetrating glaucoma surgery (NPGS) aiming to reconstruct the physiological outflow of aqueous humor by dilating the Schlemm`s canal [11]. This effect is achieved by opening the Schlemm`s canal through a trabeculo-descemetic window and by performing a 360° cannulation of the canal [12]. The Schlemm's canal is reported to be collapsed in the majority of the patients with primary open-angle glaucoma (POAG) [13]. A tension suture in the Schlemm`s canal provides permanent inward distension in order to dilate the canal [12].

Both methods, implantation of the PreserFlo™ MircoShunt as well as canaloplasty, are well-established IOP-reducing surgeries for patients with open-angle glaucoma [12, 14, 15, 16]. The purpose of this retrospective clinical study was to compare both methods concerning IOP reduction as well as reduction of local antiglaucomatous therapy, postoperative complications, and absolute or qualified success.

## Materials and methods

This retrospective observational study was conducted in the department of ophthalmology at St. Johannes Hospital, Dortmund, between 2018 and 2021. In this study, patients with moderate-to-advanced open-angle glaucoma were included and underwent either PreserFlo™ MicroShunt (group A) or canaloplasty (group B). Inclusion criteria involved the following points: IOP above 18 mmHg, mean deviation (MD) of the Humphrey visual field worse than -6 dB, cup-to-disc ratio ≥ 0.7 and IOP higher than the target IOP under either maximally tolerated topical antiglaucoma medications (two to four antiglaucoma agents) or systemic therapy (acetazolamide).

All patients provided their written consent prior to surgery. The institutional review board committee of St. Johannes Hospital approved this retrospective study (13/02/2021/Preserflo). The tenets of the Declaration of Helsinki were fully respected. All patients underwent regular follow-up examinations in our department on the day of discharge (two days after surgery), two weeks, three, six, and 12 months postoperatively.

Every patient underwent measurements of their best-corrected visual acuity (BCVA). The IOP was obtained with the Goldmann applanation tonometry. Additionally, visual field perimetry with the Zeiss Humphrey Analyzer 3 (Zeiss, Jena, Germany) using the Swedish Interactive Testing Algorithm (SITA Fast) was performed. The endothelial cell density (ECD) was evaluated with the Tomey EM-4000 specular microscope (Tomey GmbH, Nürnberg, Germany).

Criteria of absolute success were a postoperative IOP between 6 mmHg and 15 mmHg and at least a 20% reduction of IOP from baseline without any postoperative antiglaucoma agents or additional glaucoma surgeries. A "qualified success" was defined as a postoperative IOP between 6 mmHg and 18 mmHg and at least a 20% reduction of the IOP from baseline under fewer or the same number of preoperative antiglaucoma agents without additional glaucoma surgeries. The requirement of subsequent glaucoma surgery or a significant reduction of endothelial cell density (ECD) was considered a failure.

The implantation of Preserflo™ Microshunt was performed by a trained surgeon. The postoperative treatment contained an antibiotic eye drop (moxifloxacin three times daily) as well as corticosteroid eye drops (six times daily) and cycloplegics twice daily for the first week. After the first week, only the corticosteroid drops were continued, and they were gradually reduced to one drop every week.

Canaloplasty was also performed by a trained surgeon. The post-operative regimen in group A was gentamicin combined with corticosteroid eye drop solution five times daily and eye ointment once daily, replaced after one week with non-corticosteroid anti-inflammatory agents five times daily for two to three weeks.

Statistical analysis was performed with Excel (Microsoft Excel 2017, Microsoft®, Redmond, Washington, USA). The data were checked for normal distribution by using histograms. To compare the development of IOP, the number of topical agents, best-corrected visual acuity (BCVA), the mean deviation (MD) of the Humphrey visual field, the retinal nerve fiber layer (RNFL), and endothelial cell density (ECD), the dependent t-test was used. The comparison of parameters between groups A and B was performed with an independent t-test. The Kaplan-Meyer analysis was used to show the cumulative probability of absolute and qualified success.

## Results

**Figure 1:**
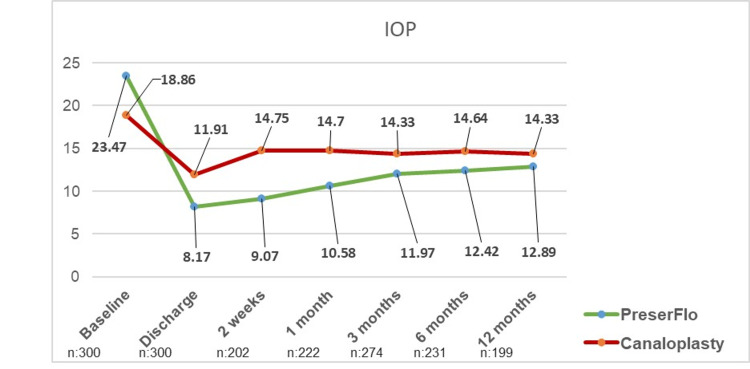
Development of intraocular pressure in groups A (PreserFlo™) and B (canaloplasty)

In total, 300 patients with moderate-to-advanced open-angle glaucoma were included in this study. These patients were equally divided into groups A (52 males and 98 females, mean age 73.30 ± 11.18 years) and B (62 males and 88 females, mean age 71.04 ± 12.89 years). 125 patients in group A suffered from POAG, whereas 21 had pseudoexfoliation glaucoma, and four patients had pigment dispersion glaucoma. In group B, 124 patients had POAG and 26 had pigment dispersion glaucoma. In group A, eight patients underwent implantation of the PreserFlo™ MicroShunt in combination with phacoemulsification. In group B, canaloplasty was combined with phacoemulsification in 81 cases. There was no statistically significant difference between the IOPs of groups A (23.47 ± 8.37 mmHg) and B (18.86 ± 5.82) at baseline (p=0.06); in group A, the IOP was significantly reduced after six months (12.42 ± 3.59 mmHg, p≤ 0.01) and 12 months (13.37 ± 3.94 mmHg, p≤ 0.01) postoperatively in comparison to baseline (23.47 ± 8.37 mmHg). In group B, there was also a reduction in IOP after six months (14.57 ±2.95 mmHG, p≤ 0.01) and 12 months (14.32 ± 3.59 mmHg, p≤0.01) in comparison to the baseline (18.86 ± 5.82 mmHg). Comparing both groups, the IOP of patients who received the PreserFlo™ MicroShunt was significantly lower than the IOP of patients receiving canaloplasty at six (p≤ 0.01) and 12 months (p=0.049, Figure 1).

**Figure 2:**
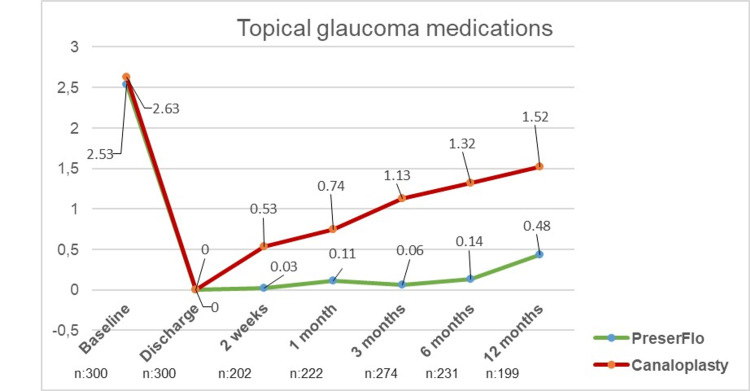
Development of the number of topical glaucoma medications needed in groups A (PreserFlo™) and B (canaloplasty)

The number of topical antiglaucoma agents did not significantly differ between groups A (2.6 ± 1.17) and B (2.6 ± 0.87) at baseline (p=0.1). Patients in group A applied significantly fewer topical agents six months (0.13 ± 0.53, p≤0.01) and 12 months (0.43 ± 0.83, p≤0.01) after surgery (baseline = 2.53 ± 1.56). There was also a significant reduction of topical agents in group B after six (1.32 ± 0.90, p≤0.01) and 12 months (1.52 ± 0.99, p≤0.01) in comparison to baseline (2.62 ± 0.87). Patients receiving the PreserFlo™ MicroShunt applied significantly fewer topical agents than patients who underwent canaloplasty (p≤0.01 six and 12 months after surgery, Figure 2).

The BCVA did not differ between both groups at the baseline (group A, 0.6 ± 0.25; group B, 0.6 ± 0.36, p=0.58). BCVA remained stable from baseline up to twelve months postoperatively in both groups (group A, p=0.15; group B, p=0.17) The mean deviation (MD) of the Humphrey visual field didn´t differ significantly between groups A (-11.8 ± 9.28) and B (-10.9 ± 7.44) at the baseline, p=0.21. In group A, MDs remained stable 12 months after surgery (-10.52 ± 8.63, p=0.17). MDs in group B showed no deterioration after 12 months (-11.72 ± 6.54, p=0.12). RNFL thickness showed either a significant difference between groups A (66.06 ± 14.44) and B (68.74 ± 10.61) at the baseline (p=0.27). The RNFL thickness remained stable after 12 months in group A (65.60 ± 12.93, p=0.40) and group B (67.41 ± 7.69, p=0.12).

Early postoperative complications in group A included ocular hypotony with choroidal detachment in 18 cases, a number that was reduced to six cases after one month and to no cases after three months postoperatively. A certain percentage of patients required surgical reformation of the anterior chamber (two patients two weeks after surgery, one patient one month, and one patient three months after initial PreserFlo™ surgery).

Five patients suffered from IOP decompensation two weeks after surgery in group A, whereas one month after surgery there were six more patients with IOP decompensation after three and six months. Bleb revision was required in one case after two weeks, in five cases after one month, in three cases after three months, and in one case after six months. Four patients underwent subconjunctival injection with mitomycin 0.02% and dexamethasone, and one patient underwent micropulse transscleral cyclophotocoagulation (mTS-CPC).

In group B, ocular hypotony with choroidal detachment occurred in two patients two weeks after surgery. The most common complication seen in group B was hyphema (15 cases). Five patients required air insufflation in the anterior chamber. Transient dissection of the Descemet membrane was seen in seven patients. Decompensation of IOP was seen in two patients after three months, in two other patients after six months, and in one patient after 12 months. Two patients in group B underwent a trabeculectomy; one patient required treatment with mTS-CPC, while the other received implantation of the Cypass Micro-Stent (before it was withdrawn from the market in 2018 [17]) due to postoperative IOP decompensation.

An overview of the postoperative complications is given in Table 1. In some cases, postoperatively, further surgery was required, and these results are presented in Table 2.

**Table 1:** Postoperative complications up to 12 months after PreserFlo and canaloplasty

	Two weeks	One month	Three months		Six months	12 months
Group A: PreserFlo™						
Ocular hypotony	18 (12%)	6 (4%)	0 (0%)	0 (0%)		0 (0%)
Leakage of wound based on the Seidel test	1 (0,66)	0 (0%)	0 (0%)	0 (0%)		0 (0%)
Choroidal detachment	18 (12%)	5 (3,33%)	0 (0%)	0 (0%)		0 (0%)
IOP decompensation	5 (3,33%)	6 (4%)	6 (4%)	2 (1,33%)		0 (0%)
Group B: canaloplasty						
Detatchment of choroidea	2 (1,33%)	0 (0%)	0 (0%)	0 (0%)		0 (0%)
Hyphema	15 (10%)	0 (0%)	0 (0%)	0 (0%)		0 (0%)
Dissection of Descemet membrane	5 (3,33%)	2 (1,33%)	0 (0%)	0 (0%)		0 (0%)
IOP decompensation	0 (0%)	0 (0%)	2 (1.33%)	2 (1.33%)		1 (0.66%)

**Table 2:** Additional surgeries after the initial surgery

	Two weeks	One month	Three months	Six months	12 months
Group A: PreserFlo™					
Removal of suture	1 (0,66)	17 (11.33%)	9 (6%)	4 (2,67%)	2 (1,33%)
Reformation of the anterior chamber	2 (1,33%)	1 (0,66)	1 (0,66)	0 (0%)	0 (0%)
Compression suture	0 (0%)	1 (0,66)	0 (0%)	0 (0%)	0 (0%)
Irrigation of the anterior chamber	1 (0,66)	1 (0,66)	1 (0,66)	0 (0%)	0 (0%)
Revision of bleb	1 (0,66)	5 (3,33%)	3 (2%)	1 (0,66)	0 (0%)
Needling	2 (1,33%)	2 (1,33%)	0 (0%)	0 (0%)	0 (0%)
Micropulse Transscleral Cyclophotocoagulation (mTS-CPC)	1 (0,66)	1 (0,66)	1 (0,66)	1 (0,66)	0 (0%)
Explantation of PreserFlo™	0 (0%)	0 (0%)	1 (0,66)	0 (0%)	0 (0%)
Group B: canaloplasty					
Irrigation of the anterior chamber	0 (0%)	5 (3.33%)	0 (0%)	0 (0%)	0 (0%)
Trabeculectomy	0 (0%)	0 (0%)	0 (0%)	1 (0,66)	1 (0,66)
Micropulse Transscleral Cyclophotocoagulation (mTS-CPC)	0 (0%)	0 (0%)	1 (0,66)	0 (0%)	0 (0%)
Ahmed-Valve	0 (0%)	0 (0%)	0 (0%)	1 (0,66)	0 (0%)
Cypass	0 (0%)	0 (0%)	0 (0%)	1 (0,66)	0 (0%)

**Figure 3:**
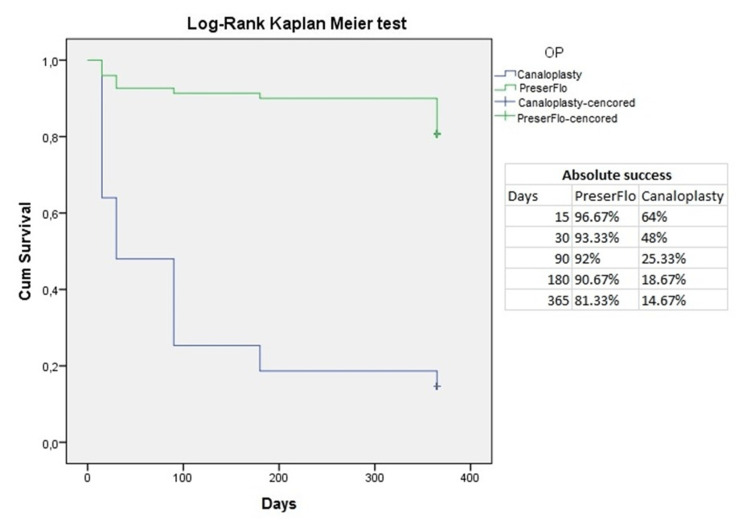
Kaplan-Meyer analysis of absolute success for PreserFlo™ and canaloplasty

**Figure 4:**
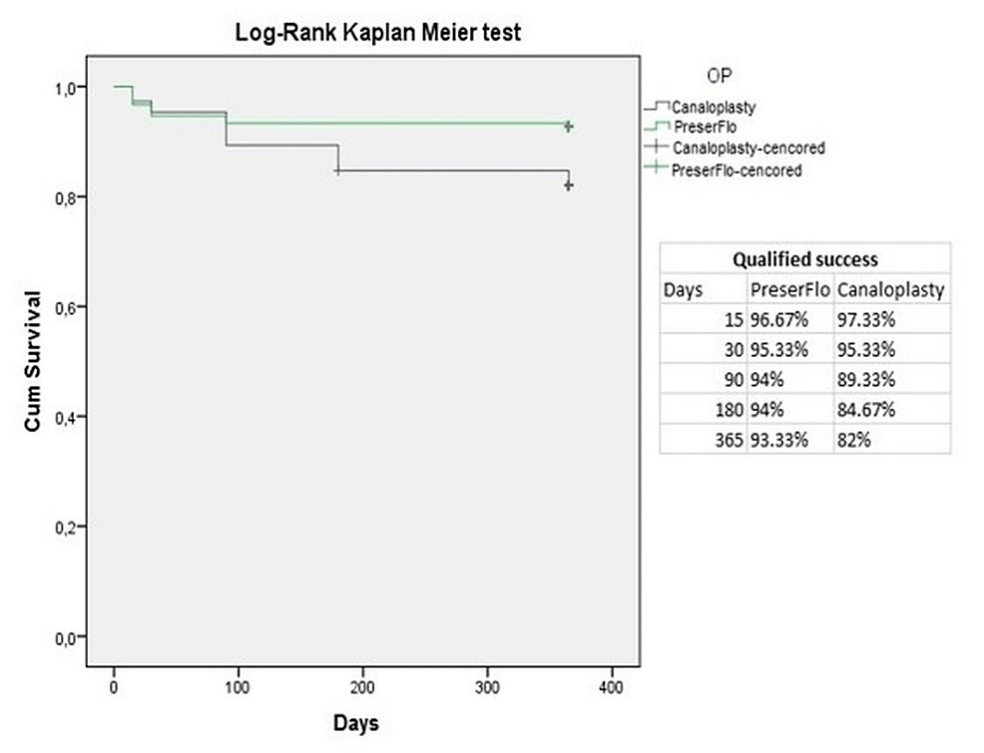
Kaplan-Meyer analysis of qualified success for PreserFlo™ and canaloplasty

The Kaplan-Meyer analysis (Figures 3, 4) showed a cumulative probability of absolute success of 96.67% in group A and 64% in group B two weeks postoperatively. One year after surgery, the cumulative probability of absolute success was 81.33% in group A and 14.67% in group B. In terms of qualified success, the cumulative probability was 96.67% in group A and 97.33% in group B two weeks after surgery. After one year, the cumulative probability of qualified success was 93.33% in group A and 82% in group B.

## Discussion

In this study, PreserFlo™ MicroShunt and canaloplasty were both able to significantly reduce the IOP and the number of antiglaucomatous agents. Vastardis et al. demonstrated a similar reduction of IOP when comparing the effects of PreserFlo MicroShunt implantation with and without Ologen. IOP in the group without Ologen decreased from 23.52 ± 5.78 to 11.56 ± 3.08 mmHg and from 26.04 ± 8.76 to 11.75 ± 3.37 mmHg in the group with the subconjunctival application of Ologen by the sixth month after surgery [4]. Batlle et al. showed as well a significant reduction of IOP from a mean of 23.8 ± 5.3 to 10.7 ± 2.8 mmHg after one year after PreserFlo implantation [18]. This effect seems to have remained stable for at least five years in the study of Batlle et al. [14]. Bhayani et al. showed in a multicenter study that the IOP decreased from 21.5 (19-28) mmHg to 13 (11-15) mmHg and 13 (11-16) mmHg at six and 12 months after implantation of PreserFlo™ [8].

In the literature, a significant reduction in IOP was also shown after canaloplasty. For example, Lewis et al. described a reduction of IOP from 24.7 ± 4.8 mmHg to 15.6 ± 4.0 mmHg at six months and 15.3 ± 3.8 mmHg one year after surgery [19], an effect that was shown to be stable three years later [20]. In a comparative study between canaloplasty and trabeculectomy, Vastardis et al. showed a reduction of IOP from 20.45 ± 7.76 to 13.87 ± 3.34 mmHg (32.17% IOP reduction by the 12th month [16].

The significant reduction of topical agents in groups A and B in our study was also in line with the literature [4, 8, 14, 16, 19, 20]. Battle et al. showed a decrease of local antiglaucomatous agents from 2.4 ± 1.0 at baseline to 0.5 ± 1.1 at year four and 0.8 ± 1.3 at year five (n=18) after PreserFlo™ implantation [14]. Vastardis et al. reported that the number of medications dropped from 2.52 ± 0.91 to 0.04 ± 0.20 after the implantation of PreserFlo with the application of mitomycin C (MMC) 0.2 mg/mL. The same authors also described a reduction in medication from 2.58 ± 0.82 to 0.16 ± 0.81 by the sixth month after implantation of PreserFlo™ with MMC 0.2 mg/ml and Ologen collagen matrix [4]. Bhayani et al did also observe a reduction in the number of medications after PreserFlo™ implantation, a number that increased over time again but remained still significantly lower in comparison to baseline [8].

Concerning canaloplasty, Lewis et al. reported a reduction in the number of medications at the twelfth month (preoperatively: 1.9 ± 1.0, 12 months postoperatively: 0.6 ± 0.9) [19]. In another study, the number of medications was 0.8 ± 0.9, compared to a baseline number of 1.8 ± 0.9 [20]. The comparison of the postoperative results in our study revealed that the PreserFlo™ MicroShunt was able to reduce IOP and the number of topical agents more efficiently than the canaloplasty. This is also reflected in the cumulative probability of absolute and qualified success: One year after surgery, the cumulative probability of absolute success was 81.33% in group A and 14.67% in group B. After one year, the cumulative probability of qualified success was 93.33% in group A and 82.00% in group B.

Pillunat et al. described a similar efficacy by comparing the implantation of the PreserFlo™ MicroShunt and the trabeculectomy concerning IOP reduction without any additional local agents [21]. The comparison of trabeculectomy to canaloplasty revealed that the trabeculectomy was able to reduce the IOP more sufficiently than the MicroShunt and with fewer additional local agents [16, 22]. Nevertheless, Matlach et al., as well as Vastardis et al., described a lower risk of postoperative complications after canaloplasty [16, 22].

The most common complication in group B was a postoperative hyphema (n = 15; 10%). Five of these patients required irrigation of the anterior chamber. Postoperative hyphema is the most known complication of canaloplasty and is the result of a rupture in the Schlemm's canal [12, 22]. However, hyphema after canaloplasty is also considered a prognostic factor for its efficacy because the presence of a hyphema proves the existence of a functional reflux between the anterior chamber, Schlemm's canal, and the vascular system [23, 24]. Another rarely described complication of canaloplasty is a transient dissection of the Descemet membrane, which occurs in approximately 5% of patients [22]. The PreserFlo™ implantation, on the other hand, can lead to, among others, the following complications: transient bulbous hypotony with choroidal detachment, bleb fibrosis, leakage of the wound based on the Seidel test, corneal edema, and hyphema [4, 14]. The higher risk of complications also explains the higher number of reoperations needed after PreserFlo™ implantation. More specifically, the most common postoperative procedures seen in our study were the surgical reformation of the anterior chamber, bleb revision, and needling. 18% of patients in group A required further interventions due to the described complications. Nevertheless, Pillunat et al. showed that the number of postoperative complications required following interventions was significantly lower than in the current gold standard, the trabeculectomy [21].

According to Zhang et al., canaloplasty offers the following advantages: no filtering bleb formation; no need for antimetabolites during surgery; rapid visual recovery; fewer complications; simple postoperative treatment; and stable postoperative IOP [11]. It is also postulated that canaloplasty is more beneficial for patients without advanced visual field damage, as an extremely low IOP is not needed to avoid further progression [12]. Furthermore, canaloplasty is approved for patients with a high risk of conjunctival scarring because there is no need for a filtering bleb, which has a high risk of failing in these patients [22].

Even though canaloplasty seems to offer a lower risk profile, one has to consider that if it fails to significantly reduce the IOP, there are only a few options to enhance the effect of the procedure [22]. In the case of trabeculectomy or PreserFlo™ implantation, bleb revision and needling are options to enhance the outflow of aqueous humor [22]. After canaloplasty, a YAG goniopuncture can be performed to achieve a greater outflow of aqueous humor through Schlemm's canal [20, 25]. If the goniopuncture remains ineffective, either a new application of topical agents or further glaucoma surgery is required [22].

Limitations

The main limitations of our study are that we collected the data retrospectively and included only a medium-term follow-up duration. Further long-term prospective studies are needed for the evaluation of the long-term efficacy and safety of PreserFlo™ Microshunt versus canaloplasty. Our results cannot be generalized to community-based glaucoma practices as the patients in this study had moderate to advanced open-angle glaucoma, which the community-based glaucoma practices would usually refer for specialist treatment.

## Conclusions

Both the PreserFlo™ and the canaloplasty are able to significantly reduce the IOP in patients with glaucoma. The cumulative probability of absolute as well as qualified success shows that PreserFlo™ was able to reduce the IOP more effectively with fewer additional topical agents in comparison to canaloplasty ab externo. Canaloplasty, on the other hand, showed hyphema as the main postoperative finding, which is also considered a good prognostic factor.

In summary, the PreserFlo™ as well as the canaloplasty offer many advantages and only a few disadvantages for patients with glaucoma. However, the respective patient’s history and individual risk profile play an important role in the decision of the glaucoma specialist regarding the most appropriate surgical treatment for each patient. Patients with a high risk of conjunctival scarring and postoperative complications may benefit more from a canaloplasty, whereas patients who need a lower average IOP and show intolerance to any topical agents may require the PreserFlo™ implantation.
